# Hydrogen Oxidation and Oxygen Reduction Reactions on an OsRu-Based Electrocatalyst Synthesized by Microwave Irradiation

**DOI:** 10.3390/ma14195692

**Published:** 2021-09-30

**Authors:** Ángela Selva-Ochoa, Javier Su-Gallegos, Pathiyammattom Joseph Sebastian, Lorena Magallón-Cacho, Edgar Borja-Arco

**Affiliations:** 1 Department of Theoretical Physics and Chemistry, Faculty of Chemistry, National Autonomous University of Mexico, Mexico City 04510, Mexico; a.selvaoa@quimica.unam.mx (Á.S.-O.); javiersugallegos@gmail.com (J.S.-G.); 2 Renewable Energy Institute, National Autonomous University of Mexico, Cuernavaca 62580, Mexico; sjp@ier.unam.mx; 3 Cátedras-CONACYT, National Institute of Electricity and Clean Energies, Cuernavaca 62490, Mexico; lorena.magallon@ineel.mx

**Keywords:** microwave synthesis, bimetallic electrocatalysts, oxygen reduction, hydrogen oxidation

## Abstract

This work presents an OsRu-based electrocatalyst synthesis, by a rapid and efficient method through microwave irradiation. The outstanding electrocatalyst shows a dual catalytic activity, demonstrating both: hydrogen oxidation and oxygen reduction reactions. The material is structural and morphologically characterized by FT-IR, X-ray diffraction, EDS, and SEM, indicating nanoparticulated Os and Ru metallic phases with a crystallite size of ∼6 nm, calculated by the Scherrer equation. The metal nanoparticles are apparently deposited on a carbonaceous sponge-like morphology structure. Its electrochemical characterization is performed in 0.5 M H_2_SO_4_ by the rotating disk electrode technique, employing cyclic and linear sweep voltammetry. Two different ink treatments have been studied to improve the obtained polarization curves. The material is also tested in the presence of methanol for the oxygen reduction reaction, showing an important resistance to this contaminant, making it viable for its use in direct methanol fuel cells (DMFCs) as a cathode and in polymer electrolyte fuel cells (PEMFCs) as an anode as much as a cathode.

## 1. Introduction

A PEMFC is an efficient and environmentally friendly power device that converts chemical energy into electrical energy through two electrochemical reactions: the hydrogen oxidation reaction (HOR) at the anode and the oxygen reduction reaction (ORR) at the cathode. Both electrochemical reactions are usually promoted by electrocatalyst materials composed of metal nanoparticles supported on carbon, whose activity becomes crucial for cell operation. These materials are usually noble metals or alloys, with nano-sized platinum particles being the most widely used metal, as it is the material that shows the highest catalytic activity towards hydrogen oxidation and oxygen reduction reactions thus far. At room temperature in an acidic medium, Pt exhibits low overpotentials, fast kinetics, and a high exchange current density (j_0_ ≈ 1 mA cm^−2^) for HOR [1]. Whereas for the ORR, the sluggish kinetics lead to high overpotentials, giving as result exchange current densities three to four orders of magnitude lower compared to the anodic reaction (j_0_ ≈ 10^−3^–10^−4^ mA cm^−2^) [2,3]. Nonetheless, Pt remains as one of the most active electrocatalysts for both reactions. However, its limited resources, high costs, and facile poisoning are still some of the remaining drawbacks of its usage. Hence, the synthesis of new electrocatalysts with dual activity as both anode and cathode in a PEMFC, to decrease Pt employment, is a challenge for the scientific community. Some materials showing this dual activity (most electrocatalysts reported in the literature focus only on one of the two reactions) are electrocatalysts based on Ir [4], Pd, Rh, Pd_x_Rh_x_ [5], Os [6], and Ru [7], where electrochemical studies are performed in an acidic medium, while a noble metal-free electrocatalyst (SnO_2_/CeO_2_) has been reported for hydrogen oxidation reactions and oxygen reduction reactions in an alkaline medium [8].

Furthermore, the use of efficient methods for the synthesis of electrocatalysts is one of the great challenges to overcome, as the advances made in this field have largely shown the requirement of long and complicated synthesis methods [9,10,11]. It is well known that conventional heating methods are slow and inefficient for energy transfer; therefore, in most cases, long synthesis times are needed (hours or days), or only organic solvents can be used as a reaction medium. On the other hand, microwave-assisted synthesis is proposed as an efficient method for heat transfer, which confers certain characteristics such as shorter synthesis times (5–30 min) and varied microwave absorbing solvent options, including water. Therefore, in the last decade, the use of microwaves as an alternative heating method for synthesis has increased [12].

In this work, the physical and electrochemical properties of an OsRu electrocatalyst synthesized by microwave irradiation are compared with a similar material obtained by a conventional heating method (refluxing) [13]. We thereby demonstrate that the use of microwaves stands as an environmentally friendly method of synthesis that confers several improvements to the whole synthetic process and to the electrocatalyst itself. For example, it is able to reduce synthetic times from 20 h to only 30 min with a null obtention of by-products and a largely improved electrocatalytic activity towards both HOR and ORR.

## 2. Materials and Methods

### 2.1. Microwave-Assisted Synthesis of OsRu

The OsRu bimetallic electrocatalyst was synthesized using an equimolar ratio of the precursors dodecacarbonyl triruthenium (Ru_3_(CO)_12_ 99%, Sigma-Aldrich, San Luis, MO, USA) and dodecacarbonyl triosmium (Os_3_(CO)_12_ 98%, Sigma-Aldrich, San Luis, MO, USA), which were mixed with 10 mL of 1,2-dichlorobenzene in an ultrasonic bath for 10 min. The mixture was placed in a polytetrafluoroethylene (PTFE) tube and subjected to microwave heat treatment using a microwave reactor (Synthos 3000, Anton Paar, NSW, Australia), maintaining a temperature of 180 °C for 30 min at 200 W. The black powders obtained were separated from the solvent, rinsed with acetone, and dried at room temperature.

### 2.2. Morphological and Structural Characterization

The structural characterization of the electrocatalyst synthesized was performed by the Fourier transform infrared spectroscopy (FT-IR) technique in a PerkinElmer-UATR-Two spectrophotometer (PerkinElmer-Mexico, Ciudad de Mexico, Mexico), and by X-ray diffraction in a Bruker D8 Advance X-ray diffractometer (Cu Kα1 radiation, 1.5406 Å, Bruker Mexicana, CDMX, Ciudad de Mexico, Mexico). The morphological and chemical composition characterization was performed by scanning electron microscopy (SEM) and energy dispersive spectroscopy (EDS), respectively, using an Ultra-High-Resolution JEOL JSM-7800F microscope (SEMTech Solutions, North Billerica, MA, USA).

### 2.3. Electrode Preparation

Electrochemical studies were performed by the rotating disk electrode technique (RDE) at 25 °C in a three-electrode electrochemical cell, comprising a working electrode (electrocatalytic ink deposited on a glassy carbon disk, Ø = 5.0 mm), reference electrode (Hg/Hg_2_SO_4_/0.5 M H_2_SO_4_; 0.68 V/NHE), and counter electrode (graphite rod). The potential values reported are referred to the normal hydrogen electrode (NHE). The 0.5 M H_2_SO_4_ used as the supporting electrolyte was prepared with 98 % H_2_SO_4_ (J.T. Baker, Fisher Scientific, Madrid, Spain) and deionized water (18.2 MΩ cm). The RDE was operated with a speed control unit (AMETEK, 616A, Princeton Applied Research, Oak Ridge, TN, USA), and the electrochemical data was acquired by a bipotentiostat/galvanostat (PINE, Wavedriver AFP2, Durham, NC, USA) commanded by AfterMath^®^ software (v 1.6.10513, Durham, NC, USA). A PolyScience (Model 9001) recirculator was used for temperature control.

For the working electrode, two different inks were prepared by mixing the electrocatalyst at a 30 wt% OsRu/Vulcan^®^ XC-72 (Cabot, Boston, MA, USA) ratio, Nafion^®^ (5% in aliphatic alcohols, Sigma-Aldrich, San Luis, MO, USA), and isopropyl alcohol (99.5%, Meyer, CDMX, Mexico) at different proportions: one for the HOR studies and the other for the ORR studies. The amounts and loadings for each ink prepared are shown in Table 1. Both mixtures were sonicated for 10 min on a sonicator (Cole-Palmer, IL, USA) and then 10 μL of the resulting ink were deposited on the glassy carbon (GC) disk electrode (geometrical surface area = 0.1963 cm^2^), previously polished on a MicroCloth polishing with alumina abrasive (5 and 0.3 µm) and rinsed by ultrasonication in deionized water. Also, 30% Pt/Vulcan XC-72 inks were prepared under similar conditions for each reaction for comparison.

### 2.4. Cyclic Voltammetry

Cyclic voltammetry (CV) experiments were done to clean, activate, and characterize the electrode surface for both reactions (ORR and HOR), with a previous purge of the electrolyte with pure N_2_ (Praxair, UHP, CDMX, Mexico) for 30 min. Potential sweeps were done for ∼48 cycles, from 0 to 0.98 V/NHE at a 20 mV s^−1^ scan rate.

An accelerated durability test (ADT) was conducted for the material in the same electrochemical cell, cycling from 0 to 0.98 V/NHE at 50 mV s^−1^ for 2000 cycles with normal air conditions. Voltammograms were recorded periodically to study the electrocatalyst degradation.

### 2.5. Hydrogen Oxidation Reaction

Once the electrode surface was activated, the electrolyte was saturated with H_2_ (Praxair, UHP, CDMX, Mexico), measuring the open circuit potential (OCP) to reach the equilibrium. Then the HOR was studied by linear sweep voltammetry (LSV) technique from the open circuit potential (OCP) (E_OC_) to 0.25 V/NHE at a 5 mV s^−1^ rate. The rotation electrode rates ranged from 100 to 900 rpm. All the measurements were repeated with a new ink application but without performing CV activation, and for platinum with previous activation.

### 2.6. Oxygen Reduction Reaction

The ORR was studied in both the absence and presence of methanol. To do so, the electrolyte was saturated with O_2_ (Praxair, UHP, CDMX, Mexico), measuring the OCP until equilibrium. LSV curves were obtained from the OCP to 0.08 V/NHE at 5 mV s^−1^. This was performed with CV ink activation and without it, ranging from 100 to 900 rpm and from 100 to 1600 rpm electrode rates, respectively. The ORR studies in the presence of methanol were executed once the studies in the absence of methanol were finished, deoxygenating the electrolyte with pure N_2_ for 30 min in between and adding absolute methanol (99.99%, J.T. Baker, Fisher Scientific, Madrid, Spain) to reach a final CH_3_OH concentration of 2.0 mol L^−1^, after which the LSV procedure was repeated. This was only done in the ink without previous activation, to obtain well-defined polarization curves by avoiding the electrochemical formation of metal oxides on the surface of the electrocatalyst. ORR studies were also performed on platinum as a reference.

## 3. Results and Discussion

### 3.1. Morphological and Structural Characterization

The detected elements and their quantities were obtained by EDS analysis; they are shown in Table 2. The results show that the OsRu electrocatalyst has a higher Ru amount than Os (with an approximated ratio of 4:1 wt% or 7:1 at% of Ru:Os). The difference between the expected and experimental molar ratio could be a consequence of the Ru_3_(CO)_12_ solubility in dichlorobenzene, which is greater than the Os_3_(CO)_12_ one. This could have caused a more efficient decarboxylation of Ru during microwave reaction, while the non-solubilized Os reagent fraction remained unreacted in the solvent.

**Figure 1:**
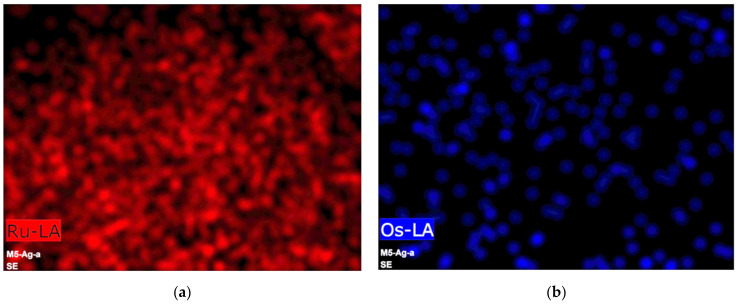

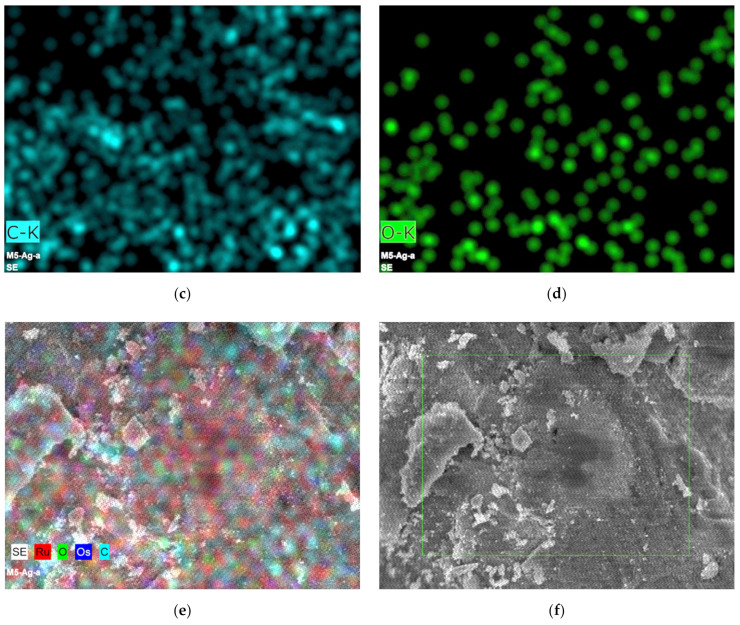
Individual elemental distribution mappings of (**a**) Ru, (**b**) Os, (**c**) C, (**d**) O, and (**e**) overlay of a (**f**) selected area in the OsRu material.

Subsequently, as part of the structural analysis, an elemental mapping of a representative portion of the synthesized material was carried out. The maps obtained (Figure 1) show the presence of Ru, Os, C, and O. It can be seen that Ru and C are distributed throughout the selected area, while Os and O appear only in some areas. Also, it can be observed that Ru, Os, and O coincide in some points. On the other hand, Figure 1e shows the superposition of all mappings, from which we can observe that most of the C is found free in many areas, and in others, it coincides with Os and O. This means that although there could be metallic oxides, the map suggests that the amounts obtained must be small. It can thus be supposed that metals are mainly in a zero-oxidation state. Furthermore, as C and O coexist in many areas, the O is presumably in the C phase.

**Figure 2:**
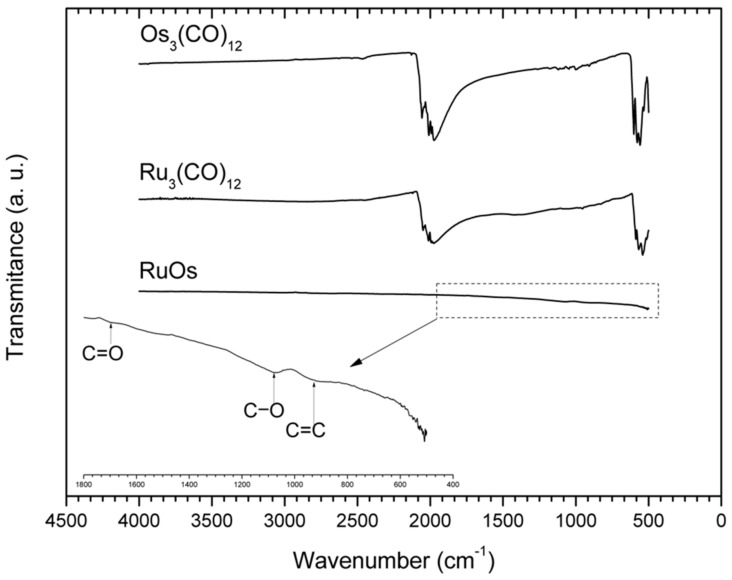
FT-IR spectra of the OsRu electrocatalyst synthesized at 180 °C by microwave radiation in 1,2-dichlorobenzene and the precursors Os_3_(CO)_12_ and Ru_3_(CO)_12_.

To confirm the absence of carbonyl groups in the material, the transmittance spectra were obtained for the precursors used and for the synthesized material. Figure 2 shows the comparative FT-IR spectra of the synthesized OsRu electrocatalyst and its precursors (Os_3_(CO)_12_ and Ru_3_(CO)_12_). For the precursors, two signals around 2000 and 500 cm^−1^ can be observed, related to the C-O stretching frequency of the carbonyl complexes (δ_C-O_) and to the carbonyl deformation modes (δ_M-CO_), respectively. For the OsRu electrocatalyst, no characteristic signals of the carbonyl groups can be seen, indicating that the metals present in the new material suffered a complete decarboxylation. In addition, by broadening the spectrum between 400 and 1800 cm^−1^, small signals appear around 900, 1100, and 1700 cm^−1^, which could be associated with C=C bending, C-O stretching, and C=O stretching, respectively [14]. The presence of the latter signals may be due to the decomposition of a small fraction of the solvent by microwave radiation, which may be part of the C phase observed.

**Figure 3:**
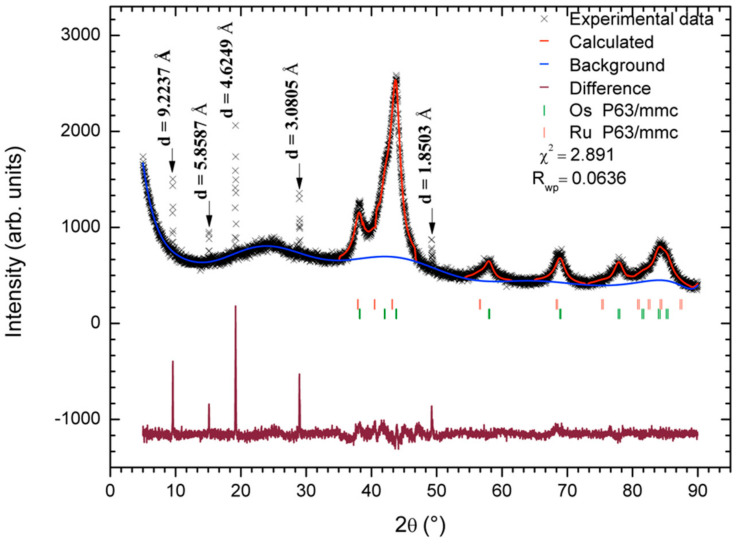
X-ray diffraction pattern of the OsRu electrocatalyst. In the plot, the black crosses represent the experimental data, the red line represents the calculated pattern, the blue line represents the background used for refinement, and the maroon line at the bottom represents the difference between the experimental and calculated pattern. The orange and green bars represent the positions of the Bragg peaks for Ru (P63/mmc) and Os (P63/mmc), respectively. In addition, for 2*θ* = 9.59°, 15.12°, 19.20°, 28.97°, and 49.24°, the distances (d) calculated in each case are presented. The goodness-of-fit parameters ($\chi^{2}$ and Rwp) are also given.

Afterward, the material was characterized by X-ray diffraction with Cu Kα1 radiation to identify the phases present. The experimental diffractogram was indexed and refined by the Rietveld method [15] using the GSAS software and the EXPGUI graphical interface [16,17]. Figure 3 shows the experimental and calculated diffractogram, the expected reflections for the found phases, and the goodness-of-fit parameters ($\chi^{2}$ and R_wp_). The carbon phase was not considered in the refinement methodology because of the inherent disorder and possible randomness of carbon layers (which is reflected in goodness-of-fit parameters). However, its distances (d) and its crystallite size were calculated. In the software, the refinement parameters were calculated in the following order: (1) the background (type 1) and scale factor, (2) the lattice parameters, (3) the phase fractions, (4) the profile parameters (type 2), and (5) the positions and thermal coefficients. The resultant values are shown in Table 3.

The indexed diffractogram indicates that the OsRu material is composed of a phase mixture of Ru, Os, and C. Based on the refinement results, the Ru phase was found in a higher proportion than the Os phase, with an approximated ratio of 3:1 for Ru:Os. Moreover, the lattice parameters indicated that the Ru cell is 7.7% bigger and the Os cell is 0.56% smaller than their cells reported [18]. These changes could be associated with atom substitutions in each phase, i.e., Os for Ru in the Ru cell, and Ru for Os in the Os cell. During the refinement, an attempt to model the substituted phases was made. However, it was not possible to achieve a successful convergence of the data. So, if these substitutions were carried out, the amounts of atoms involved might be too small to be described with a multiphase model. The crystallite size values calculated for the Ru and Os phases were 6.21 and 6.90 nm, respectively. In the calculation, the Lorentzian term obtained from the refinement was used in the Scherrer equation (with K = 1).

**Figure 4:**
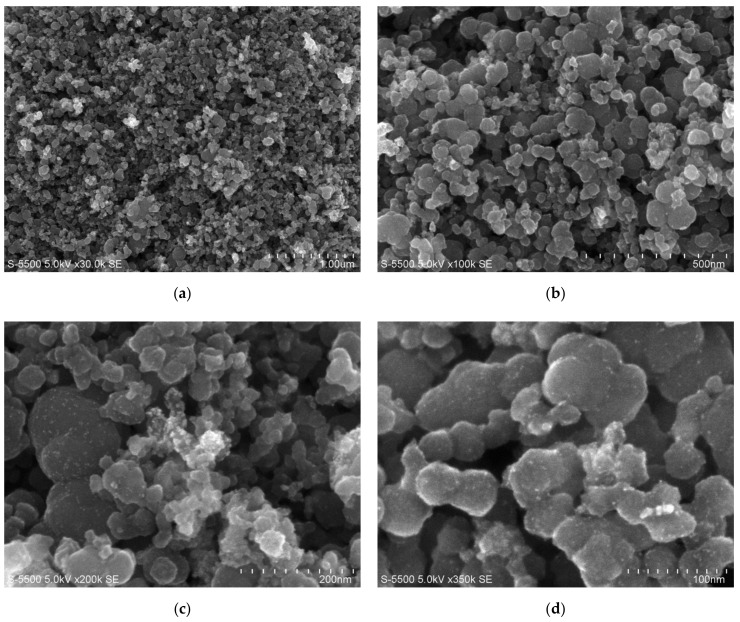
Scanning electron micrographs of the OsRu electrocatalyst synthesized at 180 °C by microwave radiation in 1,2-dichlorobenzene, at (**a**) 30×, (**b**) 100×, (**c**) 200× and (**d**) 350× magnifications.

Based on EDS signal and mapping results, the peaks at 9.59°, 15.12°, 19.20°, 28.97°, and 49.24° could be related to a phase or various phases of carbon (graphite, graphite oxide, graphite with Ru intercalated, or a mixture of these phases) [14,19,20,21,22,23,24]. Nevertheless, without further information, it is not possible to confirm that. The half-width of every peak was used in the Scherrer equation to calculate the crystallite size (with K = 1). The results in order of appearance are 129, 119, 98, 94, and 94 nm, respectively. These sizes are much larger than those obtained for the Ru and Os metallic phases, which is congruent with the differences observed in the broadness of the respective signals and with SEM micrographs shown in Figure 4.

In the scanning electron micrographs of the OsRu electrocatalyst, irregular and agglomerated particles of approximately 50–100 nm could be observed, on which smaller particles were supported. From the contrasts in the images, it could be established that the Ru and Os metallic phases were supported with a good distribution on larger carbon particles, which is congruent with the XRD results.

### 3.2. Cyclic Voltammetry

**Figure 5:**
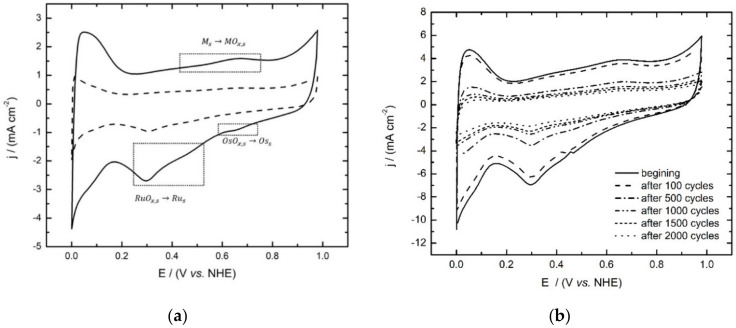
(**a**) Cyclic voltammograms of the OsRu electrocatalyst obtained for the HOR (---) and RRO (**⏤**) inks in 0.5 mol L^−1^ H_2_SO_4_ as the electrolyte at a sweep rate of 20 mV s^−1^ and (**b**) successive voltammograms for stability proofs at 50 mV s^1^ recorded at specific stages.

The cyclic voltammograms of the OsRu electrocatalyst inks are shown in Figure 5 in conjunction with stability proofs. In Figure 5a, it is shown that both inks keep hydrogen adsorption and desorption peaks from 0 to 0.1 V/NHE and the oxygen evolution in the anodic 0.8–1.0 V/NHE zone; as same as the slight anodic and cathodic peaks in the 0.4–0.6 V/NHE region ascribed to the formation and reduction of superficial ruthenium oxides and a remarkable cathodic peak observed at ∼0.2–0.4 V/NHE associated to the reduction of the oxide film formed (RuO_x,s_). The main difference may be that the ORR ink voltammogram shows a more clear oxidation and reduction zone at 0.6–0.8 V/NHE, attributed to the oxidation–reduction process of osmium metal particles (OsO_x,s_), while the HOR ink voltammogram resulted in a more Ru profile type, which may be due to the low Os at% combined with the minor electrocatalyst load on the electrode. Despite this fact, both cyclic voltammograms show the characteristic regions that confirm the presence of Os [6] and Ru [25,26].

Figure 5b represents the changes of voltammograms during ADT at different stages. It can be observed that from 100 to 500 cycles, there is a maximum decrease in current, but and after 500 cycles, the current decreases more slowly. Furthermore, it can be seen that the material is stable even after 2000 cycles.

### 3.3. Hydrogen Oxidation Reaction

**Figure 6:**
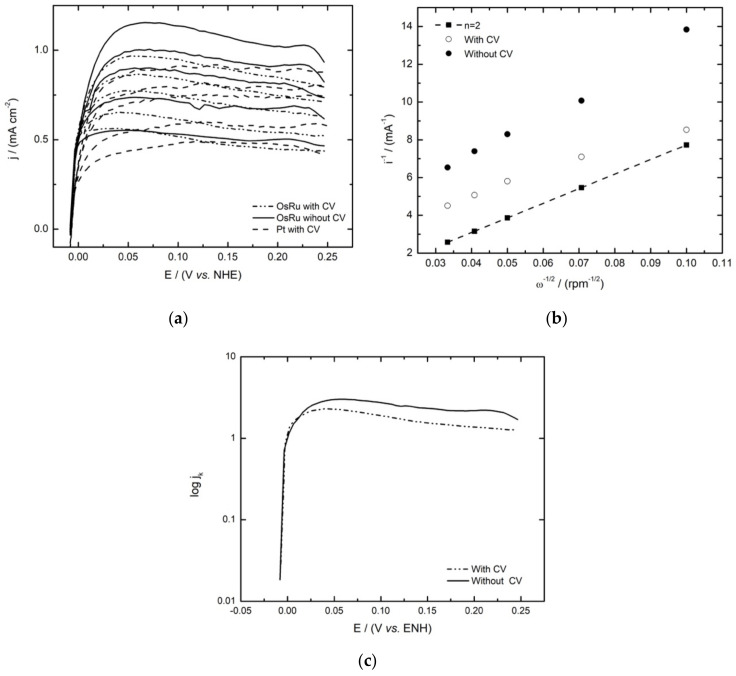
Hydrogen oxidation reaction studies with and without ink activation of the OsRu electrocatalyst in 0.5 mol L^−1^ H_2_SO_4_: (**a**) Linear sweep voltammetry polarization curves compared with 30 wt% Pt/Vulcan XC-72 ink (sweep rate 5 mV s^−1^); (**b**) Experimental and theoretical Koutecky–Levich plots; (**c**) mass-corrected Tafel plots.

Figure 6a shows the linear sweep voltammograms for the HOR electrocatalysed by the OsRu material with and without previous activation. As can be seen in both cases, the HOR starts at 0.0 V/NHE, and as the potential rises anodically, the current density starts to rise sharply, up to ∼0.04 V/NHE; after this potential, the mass transport controlled current densities are reached (*j_L_*), increasing proportionally with the rotational speed of the electrode and exhibiting a well-defined diffusional plateau.

The Koutecky–Levich theoretical plot for the two electron reaction of the HOR was obtained using Equation (1), where 1/*B* is the Koutecky–Levich slope, *n* is the number of electrons involved (2e^−^), *A* is the geometrical area of the electrode, and *F* is the Faraday constant; the values of the electrolyte kinematic viscosity (*v*), the hydrogen diffusion coefficient (*D*), and the concentration (C) in H_2_SO_4_ were considered as *v* = 0.01 cm^2^ s^−1^, $D_{H_{2}}$= 3.7 × 10^−5^ cm^2^ s^−1^, and $C_{H_{2}}$ = 7.14 × 10^−7^ mol cm^−3^, respectively [25].

(1) $$\frac{1}{B}=\frac{1}{200nAF\nu^{-1/6}D_{H_{2}}^{2/3}C_{H_{2}}}$$

By comparing the theoretical and experimental (at ∼0.16 V/NHE) Koutecky–Levich plots in Figure 6b, as expected, the experimental slopes were very close to the theoretical one. The kinetic currents (*i_k_*) obtained from the Koutecky–Levich plots were used to construct the mass-corrected Tafel curves (log *j_k_* vs. *E*) by using Equation (2), as described in the literature [27].

(2) $$i_{k}=\frac{i\;\cdot \;i_{d}}{i_{d}-i}$$

From the kinetic analysis of the Tafel plots for the HOR (Figure 6c), the exchange current density (*j*_0_), charge transfer coefficient (1 − *α*), and Tafel slope (*b*) were obtained. These electrokinetic parameters are shown in Table 4, along with those reported for Os_x_Ru_y_(CO)_n_, Ru- and Os-based electrocatalysts, and with Pt for comparison. An important aspect to highlight here is the exchange current density values, which are higher for the bimetallic materials than for the monometallic ones reported in literature, suggesting that the presence of both metals enhances the activity of the material to the HOR; this is because the presence of Os modifies Ru adsorption characteristics by shifting the d-band. In Ru, the reaction rate depends mainly on H_2_ adsorption and proton discharge from the surface [23]. In this way, Os alters the Ru–H interaction by increasing the number of protons released, resulting in a faster reaction rate and, as a result, a more significant current density.

Furthermore, the improvement is seen even when comparing this novel OsRu material with the bimetallic form synthesized by conventional methods [28], meaning that the synthesis by microwave irradiation allows us to obtain higher values of $j_{0}$ using smaller quantities of catalyst. Furthermore, compared to the Pt material at these conditions, the OsRu material shows a higher $j_{0}$ value. On the other hand, contrasting the methods of the ink study, it may seem that there are not significant differences between the activated and the non-activated ink in terms of the catalytic activity. Not so for the Tafel slope, which could be indicative of the reaction pathway followed in the HOR; according to Mello and Ticianelli [29], the material would be following a Tafel/Volmer or a Heyrovsky–Volmer mechanism for both methods, with the difference being that in the non-activated ink, the rate-determining step would be the adsorption (Tafel or Heyrovsky, Equations (3) and (4), respectively) reaction, and in the activated ink, the rate-determining step would be the discharge (Volmer, Equation (5)) reaction, which is similar for the reported electrocatalysts [28], but with a higher coverage (*θ*) of hydrogen atoms (high Tafel slope).

(3) $$H_{2}+2M⇌2MH_{ads}$$

(4) $$H_{2}+M⇌MH_{ads}+H^{+}+e^{-}$$

(5) $$MH_{ads}⇌M+H^{+}+e^{-}$$

### 3.4. Oxygen Reduction Reaction

**Figure 7:**
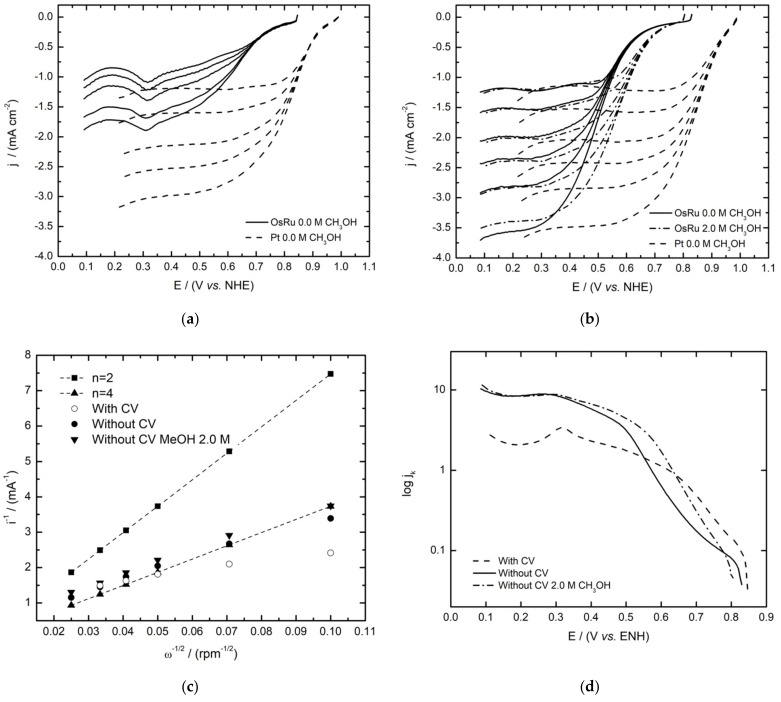
Oxygen reduction reaction polarization curves of the OsRu electrocatalyst compared with 30 wt% Pt/Vulcan XC-72 (**a**) with and (**b**) without ink activation, in the absence and presence of methanol, with its respective (**c**) Koutecky–Levich plots and (**d**) mass-corrected Tafel plots with ink activation in the absence of methanol and without ink activation in the absence and presence of methanol solution for the OsRu electrocatalyst. The electrolyte was 0.5 mol L^−1^ H_2_SO_4_ and the sweep rate 5 mV s^−1^.

As with the HOR, the OsRu material showed favorable activity towards the ORR. As can be observed in Figure 7, the difference between the ink treatment had an outstanding effect on the polarization curves acquired. In the non-activated electrode (Figure 7b), the characteristic zones for the current–potential curves of the ORR were present: the kinetic or activation control zone, the mixed control region, and the mass transfer zone, which in the case of the activated electrode (Figure 7a) by previous CV, the diffusional plateaus are not defined, as the reduction of the superficial ruthenium oxides formed previously in the CV seem to influence the currents obtained (∼0.3 V/NHE), as has been reported in other Ru catalysts [30]. So, by omitting previous CV, the ORR results seem to improve, resulting in higher current densities in the polarization curves, with a well-defined mass-transfer zone. Moreover, one of the most important features observed is that the electrocatalyst remains active towards the ORR in the presence of methanol, as can be observed in Figure 7b (non-activated ink), with just a minimum shift of the OCP and high-current densities comparable with the ones obtained in the absence of methanol, confirming the tolerance of the material to this contaminant. When comparing LSV with Pt, similar current densities are approached in the OsRu ink without previous activation.

The Koutecky–Levich analysis allowed us to identify the number of electrons involved during the ORR, with the two well-known pathways as possibilities: the four-electron mechanism by the direct reduction of O_2_ to H_2_O (Equation (6)), or the two-electron process in which hydrogen peroxide is formed as an intermediate that is subsequently reduced to water (Equation (7)).

(6) $$O_{2}+4H^{+}+4e^{-}⇌2H_{2}O$$

(7) $$O_{2}+2H^{+}+2e^{-}⇌2H_{2}O_{2}+2H^{+}+2e^{-}⇌2H_{2}O$$

The values used for the kinematic viscosity, the oxygen diffusion coefficient, and the bulk oxygen concentration were 0.01 cm^2^ s^−1^, 1.93 × 10^−5^ cm^2^ s^−1^, and 1.13 × 10^−6^ mol cm^−3^, respectively. Figure 7c shows the theoretical (for 2e^−^ and 4e^−^) and the experimental Koutecky–Levich plots for the activated and non-activated inks in the absence and presence of 2 mol L^−1^ methanol for the last one. According to the results, the experimental plots resemble the four-electron process, suggesting that in this material, O_2_ is mostly reduced to H_2_O following the direct four-electron pathway, even in the presence of methanol.

On the other hand, Figure 7d shows the mass-corrected Tafel plots for the ORR in the absence and presence of methanol for the non-activated ink and in its absence for the activated one. Through analysis, electrokinetic parameters were obtained and are summarized in Table 5 in conjunction with the OCP values for the OsRu electrocatalyst and the parameters reported in the literature for the mono [30,31] and bimetallic materials [13]. It can be observed that the OCPs are quite near one another, being slightly higher for the new electrocatalyst with CV before ORR study. In the presence of methanol (OsRu without CV before ORR), it only decreases by a small amount (~0.02 V/NHE), which is not the case, for example, for the Os electrocatalyst, which diminished by 0.16 V/NHE (almost seven times more). Furthermore, even though the other electrocatalysts did not experience a significant loss of OCPs, the OsRu material shows one of the greatest *j_0_* values in all the situations, which is one of the most important kinetic parameters, being comparable even with Pt values. Bimetallic materials show a similar behavior to the reported Ru catalyst, so the ORR could be mainly performed by Ru activity. Based on electrokinetic parameters, it is not possible to establish conclusions between inks, as the metal oxide film formed during CV affects the electrokinetic parameters, where a high Tafel slope is observed, which in turn results in low α and high j_0_ values, which could be attributed to the current imposition of the ruthenium oxide, so kinetic parameters for that ink would not be reliable. Similar behavior is observed for the metallic Ru electrocatalyst reported in the literature [7].

On the other hand, as the Tafel slope is a parameter related to the reaction mechanism [32], a value of ~118 mV dec^−1^ would indicate that the rate-determining step in the ORR corresponds to a single electron transfer, as with Pt. However, in this case, an increment of the Tafel slope can be seen, suggesting that a different ORR process was followed. With regards to the charge transfer coefficient (α), a parameter linked to the symmetry of the free energy of activation [33], the values obtained are around 0.3–0.4, which are in fact in concordance with the values reported.

Finally, this promising material allowed us to see that the electrocatalytic properties are not affected by the conjunction of the two metals, Os and Ru, furthermore, an improvement from the Os_x_Ru_y_(CO)_n_ bimetallic electrocatalyst reported. In addition, it did not require long reaction times for synthesis, nor gave different byproducts as an undesired consequence of the synthetic process. Moreover, its price is approximately $158 USD, which is a similar price to the most widely used commercial electrocatalyst (30%Pt/Vulcan; $116 USD), that could be lowered when scaling the synthesis.

## 4. Conclusions

A bimetallic OsRu-based electrocatalyst was synthesized by microwave irradiation, resulting in a nanometric glomerular structure (with a crystallite size of ∼6 nm) able to perform the HOR and/or the ORR, showing an improved catalytic activity towards these reactions in comparison to its bimetallic homologue reported in the literature. Additionally, it exhibits a significant tolerance to the presence of high methanol concentrations (2.0 mol L^−1^) during the ORR, making it a promising cathode material for DMFCs. The material is comprised of Os and Ru nanoparticles that seem to be supported on a carbonaceous structure formed during the heating process. The difference between the activated and non-activated inks showed that it is possible to improve the resulting polarization experimental curves, acquiring better diffusional plateaus, especially in the ORR. Furthermore, it is important to highlight the fact that the use of microwave energy allowed us to generate a selective synthesis method that reduced the time required for conventional thermolysis processes to only 30 min, resulting in a material with an outstanding electrocatalytic performance for the HOR and the ORR.

## Figures and Tables

**Table 1:** Electrocatalyst 30 wt%, Nafion^®^ 5 wt%, and isopropyl alcohol amounts employed for the ink preparations and their respective loadings.

Ink	Electrocatalyst	Nafion	Isopropyl Alcohol
HOR	1 mg	10 µL	90 µL
	153 µg cm^−2^	255 µg cm^−2^	---
ORR	2 mg	20 µL	20 µL
	764 µg cm^−2^	1274 µg cm^−2^	---

**Table 2:** Chemical composition of the OsRu electrocatalysts determined by EDS.

Element	Ru	Os	O
wt%	67.18	17.26	15.55
at%	38.48	5.25	56.27

**Table 3:** Crystallographic data obtained by refining the OsRu diffraction pattern: lattice parameters, % weight of each phase and crystallite size obtained from Scherrer’s equation (with K = 1), with its respective standard deviations. The space group P63/mmc with occupancy factors of 1 was used for both phases.

Phase	Lattice Parameters			wt%	Crystallite Size [nm]
	a	b	c		
Ru	2.748192	2.748192	4.469018	71.953	6.21
*σ*	0.003019	0.003019	0.006282	0.831	0.31 *
Os	2.729110	2.729110	4.311171	28.047	6.90
*σ*	0.001234	0.001234	0.002242	0.603	0.15 *

* Calculated through the uncertainty propagation law using the standard deviation of LX.

**Table 4:** Open circuit potential and electrokinetic parameters of the OsRu electrocatalyst for the hydrogen oxidation reaction (HOR) in 0.5 mol L^−1^ H_2_SO_4_, compared with similar materials reported in the literature and with 30 wt% Pt/Vulcan XC-72.

Electrocatalyst	E_OC_ (V/NHE)	b (mV decade^−1^)	(1 − *α*)	j_o_ (mA cm^−2^)
* OsRu	0.0	77	0.770	1.246
** OsRu	0.0	133	0.445	1.420
Ru [7]	0.0	49	0.601	0.113
Os [6]	0.0	43	0.416	0.031
Os_x_Ru_y_(CO)_n_ [28]	0.0	41	0.544	0.153
** 30 wt% Pt/C	0.0	70	0.85	0.55

* Without and ** with activation before HOR experiments.

**Table 5:** Open circuit potential and electrokinetic parameters of the OsRu electrocatalyst for the oxygen reduction reaction (ORR) in the absence and presence of 2.0 mol L^−1^ methanol solutions, in O_2_-saturated 0.5 mol L^−1^ H_2_SO_4_, compared with the mono and bimetallic materials reported in literature and with 30 wt% Pt/Vulcan XC-72.

Electrocatalyst	CH_3_OH [mol L^−1^]	E_OC_ (V/NHE)	b (mV decade^−1^)	*α*	j_o_ × 10^−5^ (mA cm^−2^)
* OsRu	0.0	0.830	178	0.3261	18.0
	2.0	0.805	145	0.4014	7.0
** OsRu	0.0	0.846	184	0.3158	63.8
Ru [30]	0.0	0.851	154	0.3828	26.9
	2.0	0.846	160	0.3699	29.9
Os [31]	0.0	0.761	332	0.1805	44.9
	2.0	0.598	262	0.2262	6.5
Os_x_Ru_y_(CO)_n_ [13]	0.0	0.815	129	0.4601	2.02
	2.0	0.815	132	0.4475	2.00
* 30 wt% Pt/C	0.0	0.987	103	0.5618	48.42
** 30 wt% Pt/C	0.0	0.990	93	0.6230	12.5

* Without and ** with CV before ORR studies.

## Data Availability

The data presented in this study are available on request from the corresponding author.
